# Physapubescin B inhibits tumorgenesis and circumvents taxol resistance of ovarian cancer cells through STAT3 signaling

**DOI:** 10.18632/oncotarget.19593

**Published:** 2017-07-26

**Authors:** Xiaofeng Zhao, Lu Huang, Wanwan Xu, Xiaoyan Chen, Yan Shen, Wenjie Zeng, Xiao Chen

**Affiliations:** ^1^ Department of Gynecology, Zhejiang Provincial People's Hospital, Hangzhou, Zhejiang Province, China; ^2^ Key Laboratory of Tumor Molecular Diagnosis and Individualized Medicine of Zhejiang Province, Hangzhou, Zhejiang Province, China; ^3^ Bengbu Medical College, Bengbu, Anhui Province, China; ^4^ State Key Laboratory for Diagnosis and Treatment of Infectious Diseases, Collaborative Innovation Center for Diagnosis and Treatment of Infectious Diseases, The First Affiliated Hospital, College of Medicine, Zhejiang University, Hangzhou, Zhejiang Province, China

**Keywords:** Physapubescin B, STAT3, taxol resistance, ovarian cancer

## Abstract

Ovarian cancer is the most lethal gynaecological malignancy. Recurrence and subsequent resistance to chemotherapy have become major obstacles to treating these diseases. In the present study, we showed that a natural withanolide isolated from the plant Physalis pubescens L. (Solanaceae), Physapubescin B, exhibited potent anti-tumor activity against ovarian cancer cells. Physapubescin B promoted apoptosis, induced cell-cycle arrest and inhibited invasion of ES-2 and A2780 cells. Physapubescin B treatment also resulted in suppression of the transcriptional activity of STAT3, an oncogenic transcription factor activated in many human malignancies including ovarian cancer, through disturbing the dimerization of STAT3, and thereby inhibited the nuclear translocation of Tyr705/Ser727-phosphorylated STAT3. The IL-6-stimulated activation of STAT3 and its downstream genes Cyclin D1, survivin, and Bcl-xL was also repressed by Physapubescin B. Furthermore, Physapubescin B sensitizes A2780 cells to taxol-induced cell growth inhibition *in vitro*. These findings strongly suggest that Physapubescin B has potential antitumor activity and may circumvent taxol resistance in human ovarian cancer cells through inhibition of aberrant activation of STAT3.

## INTRODUCTION

Ovarian cancer (OC) is the 9th most common cancer and the most lethal gynaecological malignancy in the female population [1–2]. It causes more than 140, 000 deaths annually in women worldwide [3]. In the past decades, more aggressive surgical techniques and the application of platinum/taxane-based chemotherapy have led to improved survival of this disease [4–5]. Nevertheless, recurrence and subsequent resistance to chemotherapy result in a poor prognosis of OCs, with a progression free survival (PFS) < 4 months and an overall survival of approximately 1 year [6]. Hence, there is an urgent need to explore alternative and more effective regimens for better adjuvant management of such patients with advanced and drug resistant OCs.

Signal transducer and activator of transcription 3 (STAT3), a member of STAT family, functions as a transcriptional factor which modulates the expressions of genes involved in a variety of physiological and pathological processes, such as inflammation, embryonic development and tumorgenesis [7–8]. In normal tissues, STAT3 is latent and predominantly located in the cytoplasm. In response to stimulation of cytokines, such as IL-6, IL-10 and vascular endothelial growth factor (VEGF), STAT3 is phosphorylated at Tyr705 and Ser727, and then translocates to the nucleus followed by dimerization and binding to DNA to promote transcriptions of its target genes [9–10]. Constitutive activation of STAT3 is causally linked to tumor development and progression in various types of solid malignancies, including head and neck cancer, myeloma, prostate cancer, breast cancer, colon cancer, and ovarian cancer [11–14]. Particularly, it has been observed in 94% of OCs and correlates with recurrence and poor prognosis [15]. Recent studies have shown that aberrant STAT3 activation was closely related with cell growth, cell cycle progression, invasion and drug resistance of ovarian cancer cells [3, 16–18]. These findings indicate that STAT3 may represent a promising molecular target of gene therapy for OCs.

Natural products from traditional Chinese herb serve as a huge library for modern drug discovery and development [19]. Growing evidences indicate that some naturally occurring withanolides, a group of steroids comprised of 28 carbon atoms, exhibited potential anti-tumor activities by inducing apoptosis or cell cycle arrest [20–21]. Physapubescin B is a newly isolated natural withanolide derived from the plant Physalispubescens L. (Solanaceae) [22]. A recent study showed that it exhibited potent activities against human prostate cancer *in vitro* and *in vivo* [23]. However, whether it has any effects on OCs remains unknown. In the present study, we explored the cytotoxic effects of Physapubescin B against OC cells and checked the involvement of STAT3 signaling in this process.

## RESULTS

### Physapubescin B inhibited OC cells growth

**Figure 1:**
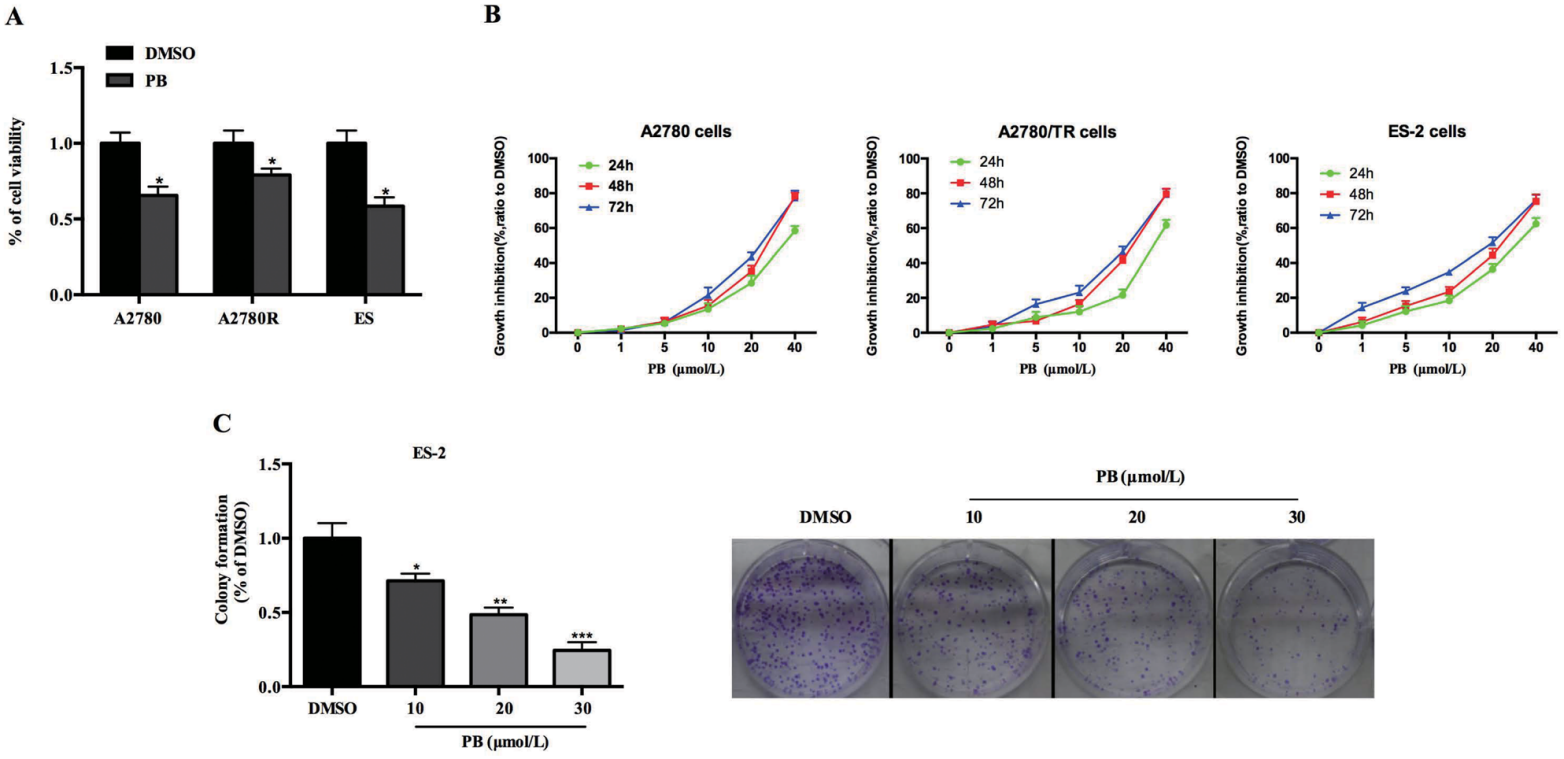
Effects of Physapubescin B on OC cell growth **(A)** Cell viabilities of A2780, A2780R and ES-2 cells were determined by CCK8 assays, 24 h after 20 μmol/L Physapubescin B were added into the cultures. **(B)** Dose effects of Physapubescin B on cell proliferation. Different dosages of Physapubescin B were added into cell cultures as in (A), and cell viabilities were assessed at each time points as indicated. **(C)** Effects of Physapubescin B on colony formation abilities of ES-2 cells. Different concentrations of Physapubescin B were added into soft agar medium before ES-2 cells were seeded in. The number of colonies were calculated 72 h after cells had been seeded (left). The representative pictures were exhibited (right, 40×). Data are presented as mean±SD. *P<0.05, **P<0.01, ***P<0.001. All experiments were repeated at least three times independently.

In a recent study, data showed that OC cell line SKOV3, with a IC50 of 6.63 ± 2.13 μM, was also sensitive to Physapubescin B in spite of prostate cancer cells [23]. We attempt to explore the cytotoxic effects of Physapubescin B on OC cells. In accordance with the previous report, Physapubescin B exerted apparent influences on OC cell lines, including A2780, A2780/TR (taxol-resistant A2780 cells) (Figure 1A). Then we treated ES-2 and A2780 Cells with increasing concentrations of Physapubescin B over the course of 72 hours. CCK8 assays indicated that Physapubescin B induced cell growth arrest of ES-2 and A2780 cells in a dose dependent manner (Figure 1B). As a control, the normal ovarian cell line, HOSE was more resistant to PB treatment (Supplementary Figure 1). Additionally, colony-forming assay showed that Physapubescin B repressed the colony generation ability of ES-2 cells in a dose dependent manner (Figure 1C). Those results suggested Physapubescin B may serve as a candidate that can inhibit OC cell growth.

### Physapubescin B induced cell-cycle arrest of OC cells

**Figure 2:**
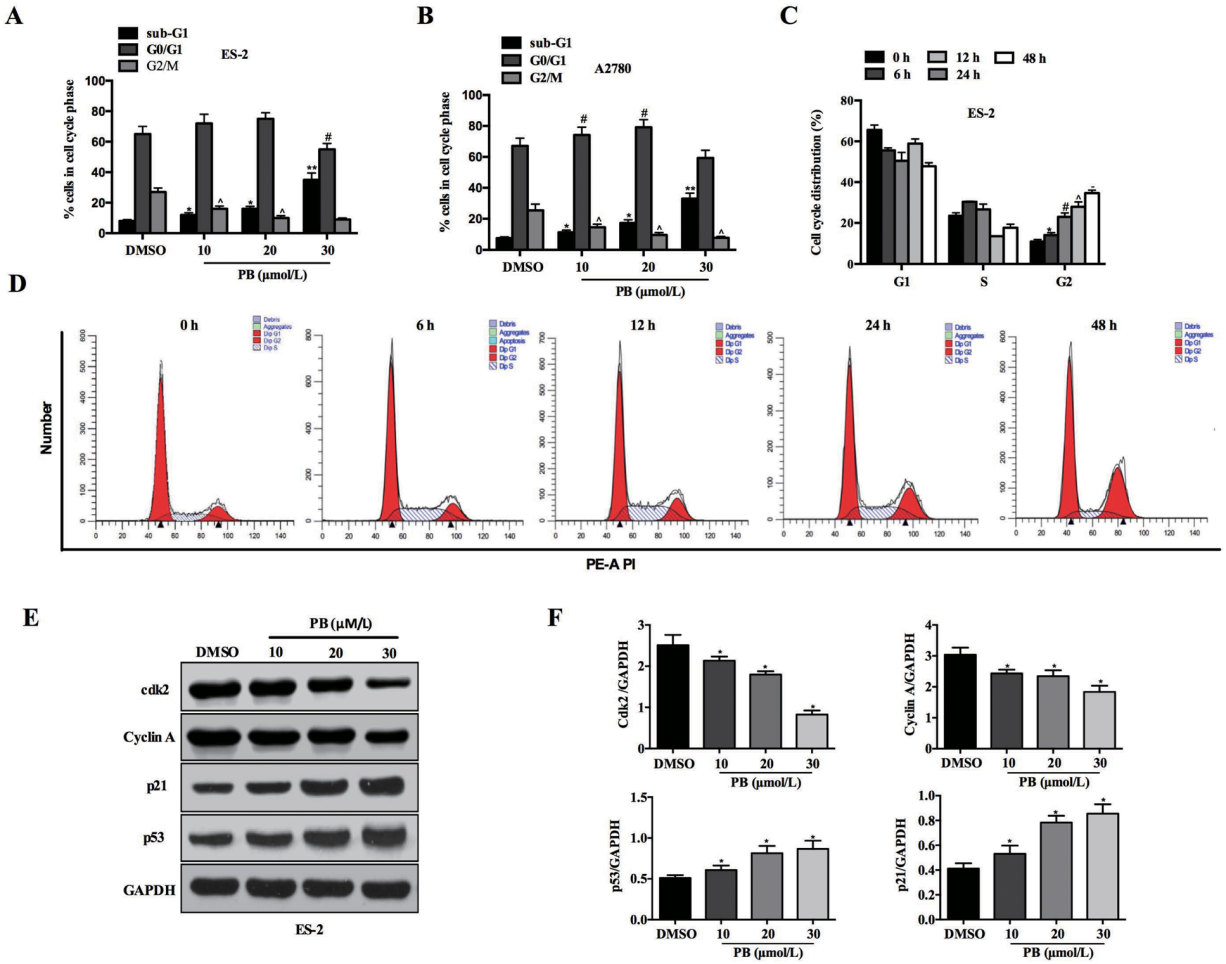
Effects of Physapubescin B on cell-cycle progression **(A** and **B)** Cell cycles were checked by PI staining, 24 h after Physapubescin B with different concentrations as indicated were added into ES-2 and A2780 cell cultures. **(C** and **D)** ES-2 cells were treated with 20 μmol/L Physapubescin B as in A, and cells cycles were analyzed by PI staining at each time points as indicated. (C) Statics results and (D) representative pictures. **(E)** ES-2 cells were treated as in A, cells were harvested for protein gel analysis. **(F)** Statics results of (E). Data are presented as mean±SD. *P<0.05, **P<0.01. All experiments were repeated at least three times independently.

To investigate the mechanism underlying the growth arrest induced by Physapubescin B, we treated ES-2 and A2780 cells with incremental concentrations of Physapubescin B, and analyzed the cell cycle with Prodium Iodide (PI) staining. Along with the increase of Physapubescin B concentration, G2-M portion declined gradually in contrast to a markedly increase distribution of sub-G1 (Figure 2A and 2B). Then we checked the time effect by treating ES-2 cells with Physapubescin B for different times. 6 h and 12 h treatment resulted in cell-cycle arrest at G0-G1 phase, whereas 24 h administration led to apoptosis indicated by a significant increase of hypodiploid cells (<2N DNA) (Figure 2C and 2D). Western blot assay showed that Physapubescin B treatment induced expression of cell-cycle regulatory genes, including p53, p21, whereas CDK2 and Cyclin A were decreased (Figure 2E and 2F), confirmed the effect of Physapubescin B on inducing cell-cycle arrest of OC cells. These results indicated that Physapubescin B could lead to G0-G1 cell-cycle arrest, at least in part, by modulating cell cycle regulatory proteins.

### Physapubescin B induced apoptosis of ES-2 cells

**Figure 3:**
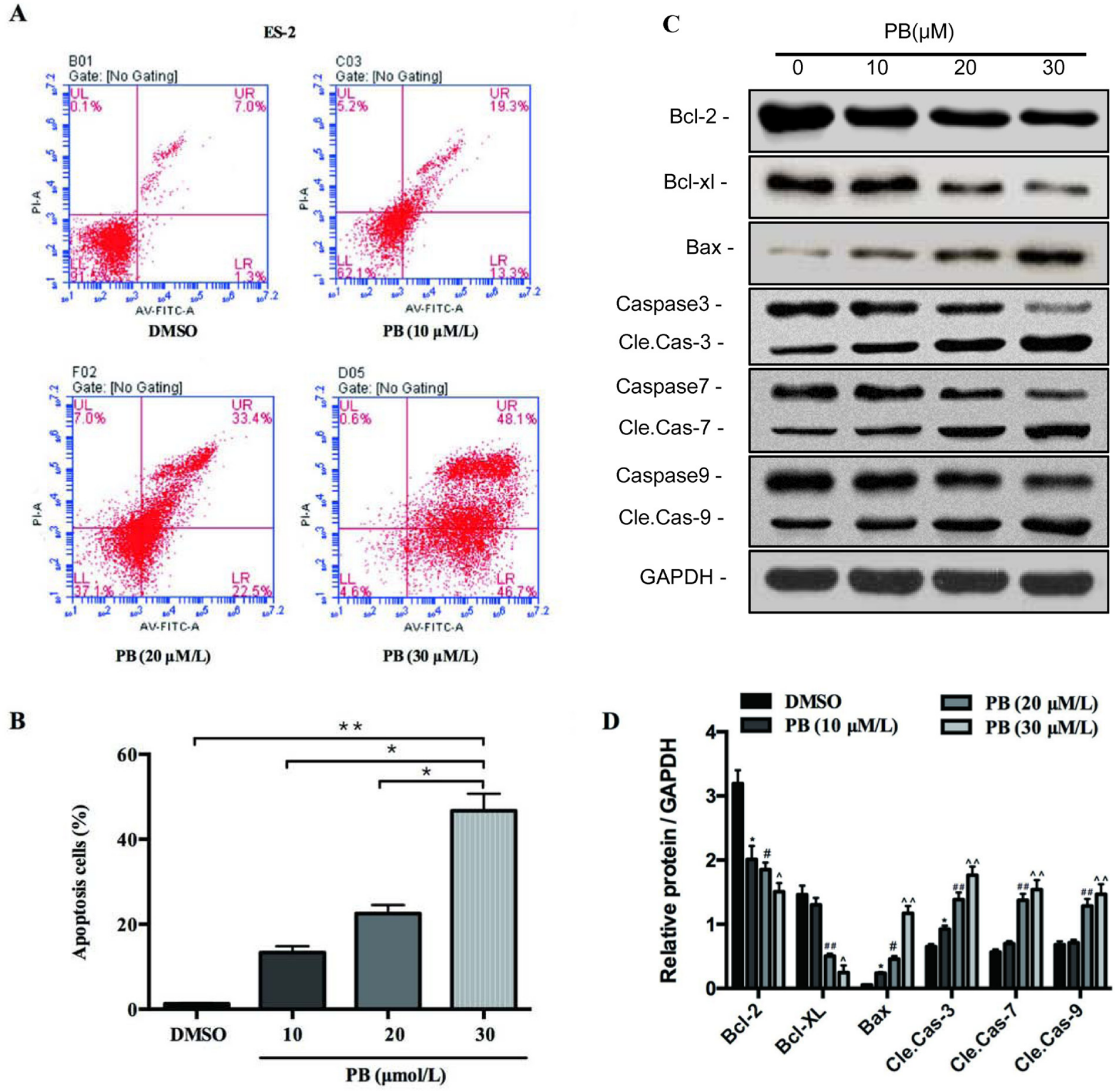
Physapubescin B induced apoptosis of OC cells **(A** and **B)** Apoptosis of ES-2 cells treated by different concentrations of Physapubescin B as indicated for 24h were analyzed by FACS. (A) Representative pictures and (B) stastics results. **(C)** ES-2 cells were treated as in A, cells were harvested for protein gel analysis. **(D)** The densitometric analyses results of (C). Data are presented as mean±SD. *P<0.05, **P<0.01. All experiments were repeated at least three times independently.

Our previous results showed that Physapubescin B may also cause cell death as shown in Figure 2C. Further experiments were designed to verify this possibility. ES-2 cells were treated with 10, 20, 30μM of Physapubescin B or left untreated as control. Treatment with 10μM of Physapubescin B induced apoptosis (13.3 vs. 1.3%), and 20 and 30μM further elevated the percentage of Annexin-V positive cells to 55.9% and 94.8% respectively (Figure 3A and 3B). We further analyzed apoptosis-associated gene expressions by Western blot. Physapubescin B treatment induced the expression of Bax, a key pro-apoptotic member of the Bcl-2 family. Accordingly, Bcl-2 and Bcl-xL, the other two members of the same family which promote cell survival, were decreased by such treatment (Figure 3C). Caspases are cysteine proteases that are commonly used to demonstrate the process of cell apoptosis. The activation of Caspases are usually characterized by an increase of the cleavage of the proteins. Treating the ES-2 cells with Physapubescin B also activated caspase-9, -3 and -7, and Poly(ADP-ribose) polymerase (PARP), demonstrated by an increase in the cleaved form of those proteins (Figure 3C). The expressions of Bax, cleaved caspases and PARP were quantified using densitometry analysis (Figure 3D). Collectively, those results suggested Physapubescin B could induce apoptosis of OC cells by activating caspases and Bcl-2 family proteins.

### Physapubescin B inhibits OC cell migration and invasion

**Figure 4:**
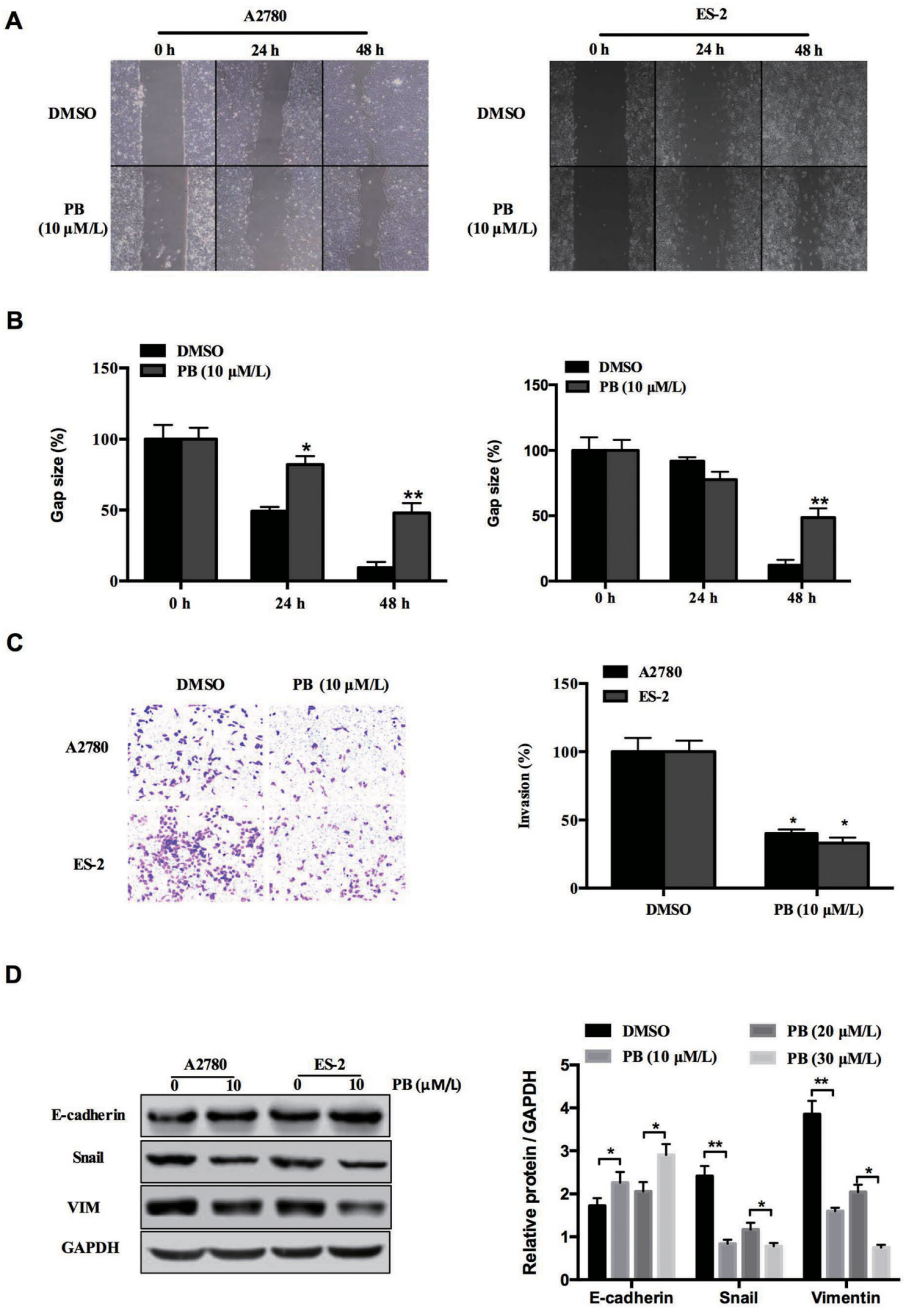
Physapubescin B inhibited migration of OC cells **(A)** Would healing assay of A2780 and ES-2 cells treated with 10 μmol/L Physapubescin B. Pictures were captured at indicated time points. **(B)** Statics results of (A). **(C)** Transwell assay of A2780 and ES-2 cells treated as in (A). Pictures were captured 48 h after seeding (left). Statics results (right panel). **(D)** A2780 and ES-2 cells were treated as in (A). Cells were harvested 48 h later, and then subjected to western blot analysis. Right panels represent the results of densitometric analyses. Data are presented as mean±SD. *P<0.05, **P<0.01. All experiments were repeated at least three times independently.

To assess the effect of Physapubescin B on the motility of ovarian cancer cells, wound-healing migration and Transwell cell invasion assays were performed by incubation of ES-2 and A2780 cells with 10 μmol/L Physapubescin B for 24 hours. Physapubescin B treatment resulted in significant inhibition of cell migration and invasion compared to untreated cells (Figure 4A, 4B and 4C). We further analyzed the expressions of genes in the regulation of epithelial-mesenchymal transition (EMT). Protein gel analysis showed that the expression of mesenchymal marker, Snail and vimentin (VIM), was down-regulated, while the epithelial marker E-cadherin expression was up-regulated in both ES-2 and A2780 cells (Figure 4D). Taken together, these results supported the conclusion that Physapubescin B inhibits OC cells migration and invasion.

### Physapubescin B inhibits STAT3 activation in OC cells

**Figure 5:**
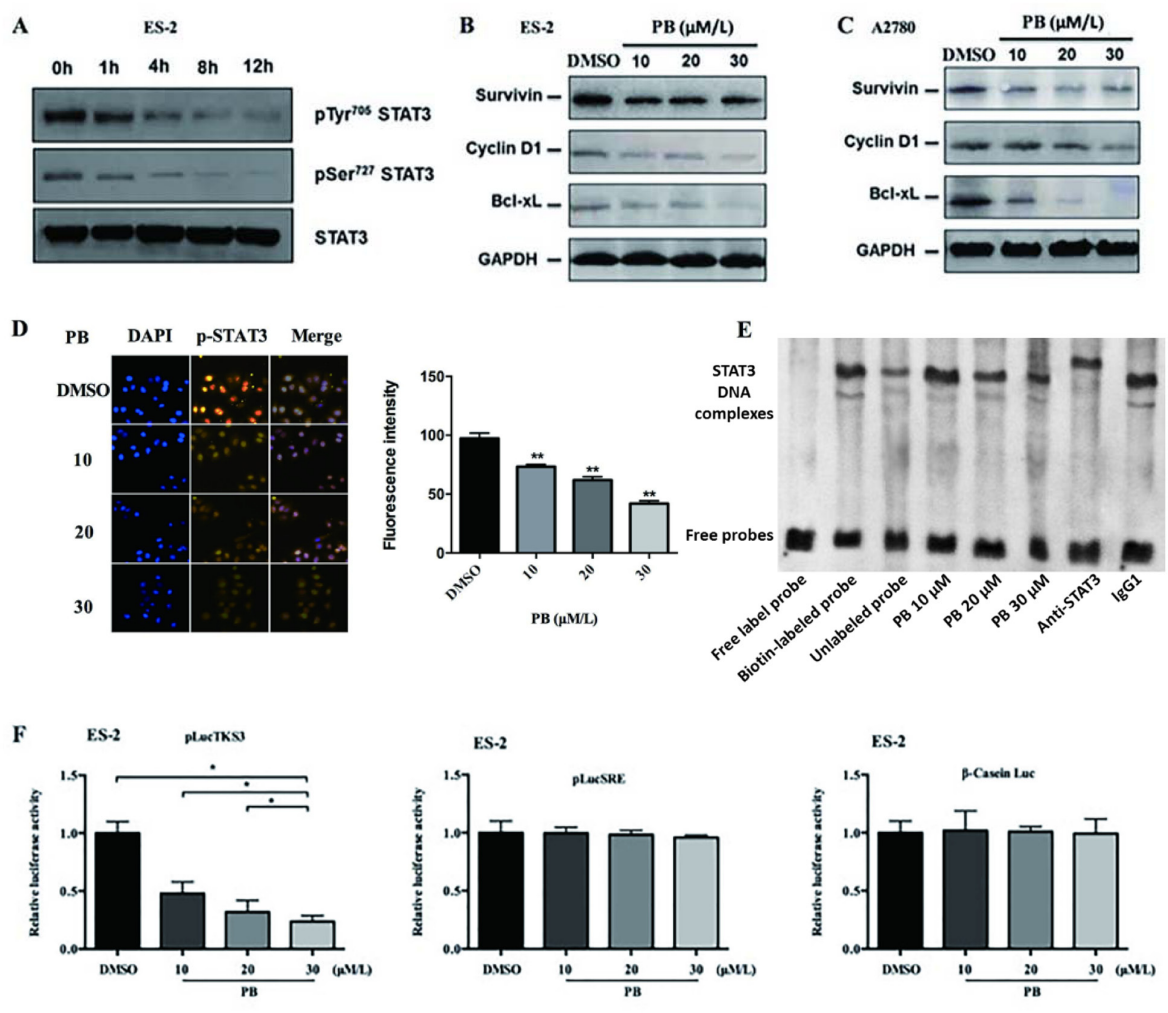
Physapubescin B inhibited STAT3 signaling in OC cells **(A)** ES-2 cells were treated with 20 μmol/L Physapubescin B. Cells were harvested at each time points as indicated and then analyzed by western blot. **(B** and **C)** ES-2 (B) and A2780 (C) cells were treated with Physapubescin B of different concentrations as indicated. Cells were harvested 24 h later and then subjected to western blot analysis. GAPDH served as a loading control. **(D)** ES-2 cells were treated with an increase amount of Physapubescin B as indicated for 24 h. Then, cells were fixed and subjected to immunofluorescence (IF) analysis. The right panels represent the results of densitometric analyses. **(E)** *In vitro* EMSA assays. Purified STAT3 protein were incubated with oligonucleotides containing the conserved STAT3 binding sites and an increase amount of Physapubescin B as indicated. **(F)** Luciferase reporter activities in cytosolic extracts prepared from ES-2 cells transiently cotransfected with the Stat3-dependent (pLucTKS3), or the Stat3-independent (pLucSRE or β-casein promoter-driven Luc) luciferase reporters together with a plasmid expressing the v-Srconcoprotein, and untreated (0.05% DMSO) or treated with different concentrations of Physapubescin B as indicated for 24 h. Data are presented as mean±SD. *P<0.05, **P<0.01. All experiments were repeated at least three times independently.

Recent studies have shown that aberrant STAT3 activation was closely related with cell growth, cell cycle progression and invasion of ovarian cancer cells [16]. Since we have demonstrated that Physapubescin B inhibited growth, cell-cycle progression, migration and invasion of OC cells, we hypothesized that it may regulate STAT3 activation in OC cells. To verify this assumption, we treated ES-2 and A2780 cells with increasing concentrations of Physapubescin B and analyzed the phosphorylated STAT3 protein levels. Physapubescin B treatment decreased Tyr705 and Ser727 phosphorylated STAT3 levels in a dose dependent fashion, without altereing the total STAT3 protein levels in ES-2 cells (Figure 5A). To further elucidate the underlying mechanisms for the induction of growth arrest and apoptosis by Physapubescin B, we detected the known targets of STAT3, including cyclin D1, Bcl-xL and survivin proteins. Protein gel blot analysis showed that treatment with Physapubescin B resulted in a decrease of cyclin D1, Bcl-xL and survivin proteins in ES-2 and A2780 cells as well (Figure 5B and 5C). Consistently, immunofluorescent assay showed that Physapubescin B treatment inhibited the nuclei translocation of STAT3 in ES-2 cells (Figure 5D). Additionally, Physapubescin B directly disturbed the DNA-binding process of STAT3 *in vitro* (Figure 5E). Luciferase reporter assay further indicated that Physapubescin B inhibited STAT3-dependent, TKS3 luciferase activity, but not STAT3-independent, SRE and β-casein luciferase activities (Figure 5F). Overexpression of STAT3 increased cells numbers and reduced apoptosis in the context of PB treatment (Supplementary Figure 2A, 2B and 2E). In concert, phosphorylated STAT3 levels were elevated by transfection of a STAT3 expressing vector (Supplementary Figure 2C and 2D). Conclusively, these results suggested that Physapubescin B could restrain STAT3 activity in OC cells and the growth inhibition effect of Physapubescin B on OC cells was exerted, at least partially, through STAT3 signaling pathway.

### Physapubescin B inhibits IL-6 mediated STAT3 activation in taxol-resistance A2780 cells

**Figure 6:**
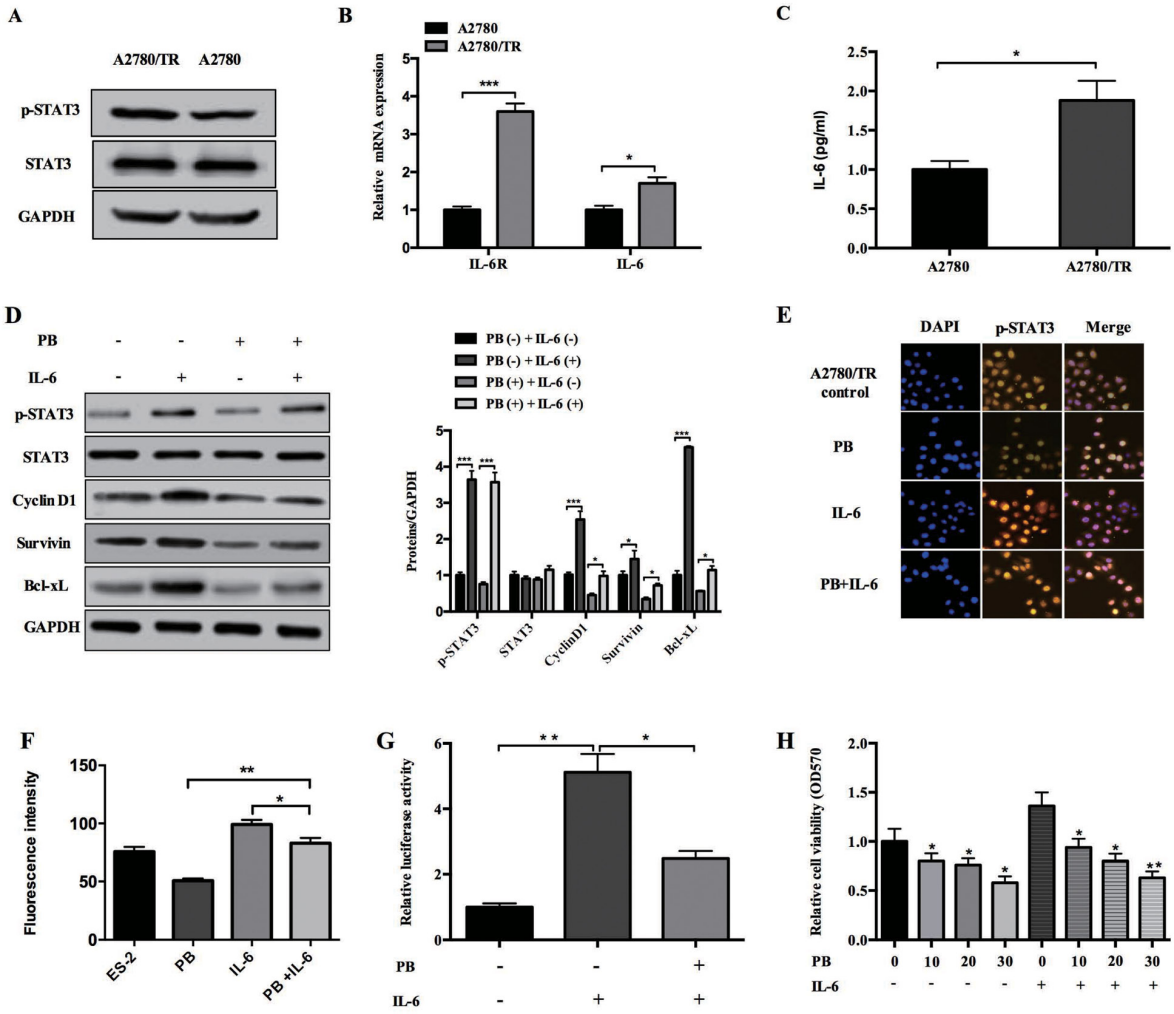
Effects of Physapubescin B on *taxol-resistance A2780 cells* **(A)** Whole cell lysates of A2780 and A2780/TR cells were subjected to western bolt analysis. **(B)** Total RNA were extracted from A2780 and A2780/TR cell cultures and then the mRNA level of IL-6 and IL-6R were determined by qPCR. Data were normalized to GAPDH. **(C)** Supernatants collected from A2780 and A2780/TR cell cultures were subjected to ELISA analysis. **(D)** A2780/TR cells were left untreated (0.05% DMSO), or treated with IL-6 (10 ng/mL), Physapubescin B (10 μmol/L), or both for 24 h. Cells were then harvested for western blot analysis. The right panels represent the results of densitometric analyses. **(E)** A2780/TR cells were treated as in (D). Cells were then fixed and subjected to IF analysis. **(F)** The results of densitometric analyses of (E). **(G)** A2780/TR cells were transfected with the pLucTKS3 luciferase reporters. 24 h later, these cells were left untreated, or treated with IL-6 (10 ng/mL), or IL-6 (10 ng/mL) and Physapubescin B (10 μmol/L). Luciferase activities in the cytosolic extracts prepared from these cells 48 h after transfection were determined by a reporter system. **(H)** A2780/TR cells were treated with different concentrations of Physapubescin B as indicated, either with IL-6 (10 ng/mL) or not. 48 h later, cell viabilities were determined by MTT assays. Data are presented as mean±SD. *P<0.05, **P<0.01. All experiments were repeated at least three times independently.

Elevated IL-6 and IL-6-mediated activation of STAT3 have been reported to cause chemoresistance to several chemotherapeutic drugs, like cisplatin and taxol, in various cancers including OCs [17, 24–25]. In accordance with Young-Ah Suh’s report, we observed increased phosphorylated STAT3 levels in taxol-resistance A2780 cells (A2780/TR) compared to non-resistance A2780 cells (Figure 6A). qPCR analysis showed that the mRNA levels of IL-6 and IL-6 receptor were also up regulated in A2780/TR cells (Figure 6B). We also quantified IL-6 protein levels in the supernatant of A2780 and A2780/TR cultures. Consistently, the secretion of IL-6 by A2780/TR cells was almost twice of that by normal A2780 cells (Figure 6C). Since Physapubescin B has been shown to inhibit constitutive STAT3 activation, we wonder whether it could repress IL-6-mediated STAT3 activation. Protein gel blot assay showed that IL-6 stimulation resulted in an increase of phosphorylated STAT3 level in A2780/TR cells, and this increase was dampened by Physapubescin B (Figure 6D). The IL-6-induced nuclei intensity of STAT3 was also inhibited by Physapubescin B treatment in A2780/TR cells (Figure 6E and 6F). Luciferase reporter assay showed that IL-6 increased STAT3 luciferase activity upto 5 folds of the control, whereas the addition of Physapubescin B declined the luciferase activity by 50-60 percent compared to IL-6 treatment alone (Figure 6G). IL-6 mediated proliferation of A2780/TR cell was also impeded by Physapubescin B in a dose dependent manner (Figure 6H). Conclusively, these results further confirmed that Physapubescin B could inhibit STAT3 activation not only in normal OC cells but also in taxol-resistance OC cells.

### Physapubescin B elevates sensitivity of taxol-resistance A2780 cells to taxol

**Figure 7:**
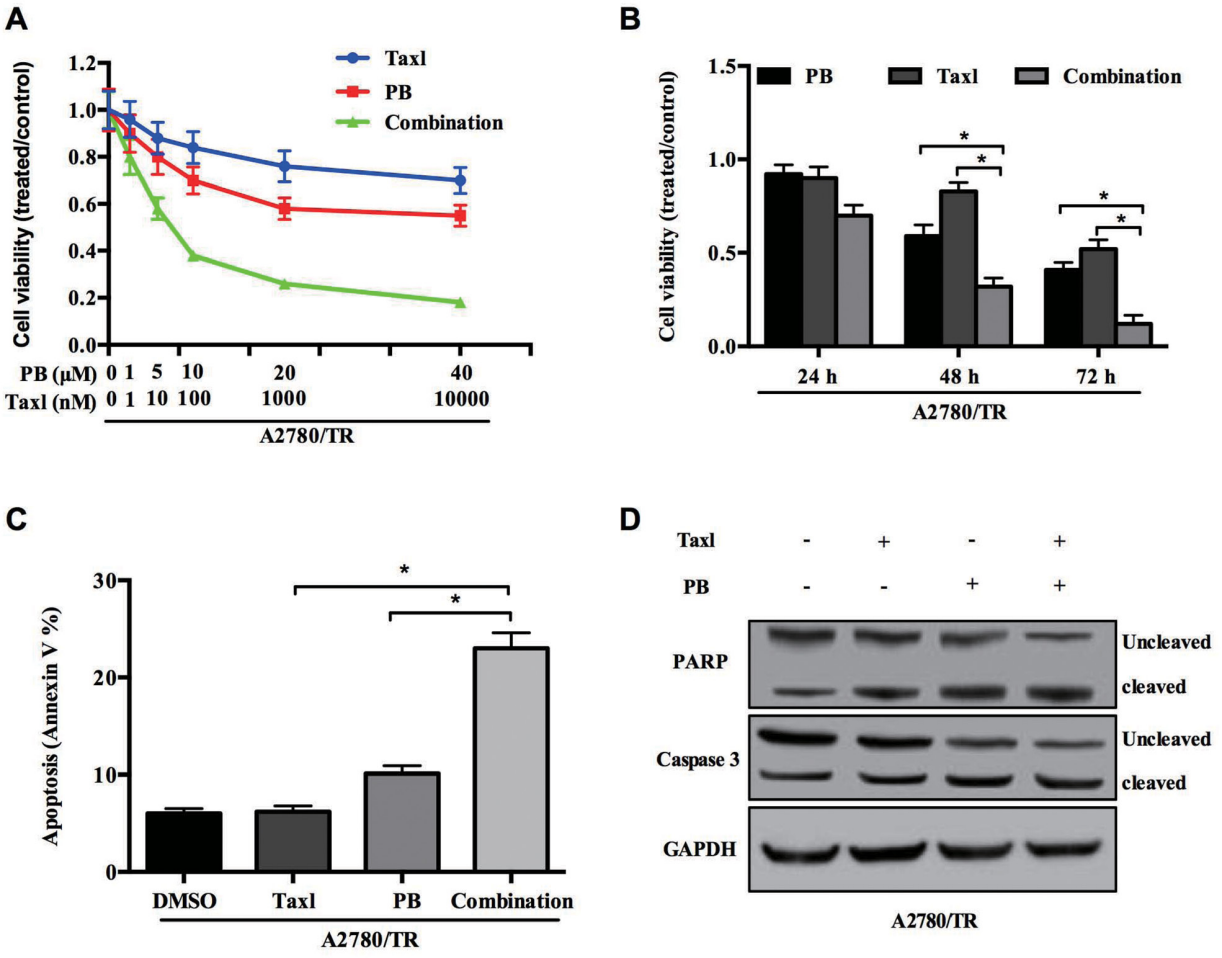
Physapubescin B resensitize taxol resistant OC cells to taxol treatment **(A)** A2780/TR cells were treated with Physapubescin B, or taxol, or both of them in different concentrations as indicated. Cell viabilities were determined 48 h later. **(B)** A2780/TR cells were treated with Physapubescin B (5 μmol/L), or taxol (10 nmol/L), or both of them. Cell viabilities were determined at each time points as indicated. **(C** and **D)** A2780/TR cells were left untreated (0.05% DMSO), or treated with Physapubescin B (5 μmol/L), or taxol (10 nmol/L, or both of them for 24 h. Cells were then collected for FASC analysis (C) and western blot analysis (D). Data are presented as mean±SD. *P<0.05. All experiments were repeated at least three times independently.

As our previous result indicated that Physapubescin B inhibited proliferation of chemoresistance OC cells besides normal OC cells, we wonder whether it could sensitize A2780/TR cell to taxol treatment. We treated A2780/TR cells with different concentrations of taxol and Physapubescin B either respectively or in combination. As predicted, A2780/TR was not sensitive to taxol, but combination of taxol with Physapubescin B significantly decreased cell viability. Tow-way ANOVA analysis indiated that there was a significant synergy effect between PB and taxol treatment. Moreover, these efficacy presented in a dose-dependent manner (Figure 7A). To assess the time-dependent effect, we treated A2780/TR cells with 10 nM taxol together with 5 μM Physapubescin B for 24, 48, 72h and analyzed the cell numbers with CCK-8. As shown in Figure 7B, the efficacy of two drugs in combination was better than any single one (Figure 7B). Next, we checked whether the synergistic effects of Physapubescin B and taxol extended to the induction of apoptosis of A2780/TR. Cells were treated with Physapubescin B and taxol either respectively or in combination for 36 h and the percentages of apoptotic cells was determined by Annexin V staining. Physapubescin B addition increased taxol-induced apoptosis from 7.4% to 32.3% (Figure 7C). In accordance with the flowcytometry analysis, the active forms of Caspase 3 and PARP increased in A2780/TR cells treated with both of the drugs (Figure 7D). These results indicated that inhibition of STAT3 activity by Physapubescin B could significantly elevated the sensitivity of taxol-resistance OC cells to taxol through enhancing apoptosis.

## DISCUSSION

Withanolides are a group of polyoxygenated C28-ergostane lactones or lactols. Previous studies have shown that it exhibits cytotoxic and immunomodulating activities [26–27]. Physapubescin B is a recently isolated withanolide from the plant Physalispubescens L. (Solanaceae), which was mainly cultivated in north China [22]. Wanjing Ding *etal*. showed that it has potential cytotoxicity to various types of tumor cells, including PC3, MDA-MB-231, HepG2, SKOV3, Du145 and RWPE-1. Among those cells, SKOV3, a commonly used OC cell line was referred to be fragile in response to Physapubescin B treatment [23]. Results in the present study showed that Physapubescin B exhibited anti-tumor activity against OC cells. More importantly, it manifested obvious effect on chemoresistance OC cells. Physapubescin B treatment overcame the chemoresistance of A2780/TR to taxol, a first line chemotherapeutic drug for OCs in clinic. We further unveiled that the underlying mechanism was by inhibiting activation of STAT3, a transcription factor frequently found to be aberrantly activated in OCs. These results add new insight to the efficacy of Physapubescin B and the mechanism how it exerts anti-tumor activity.

Constitutive activation of STAT3 is closely associated to a wide range of both solid and liquid tumors [28]. Particularly, it has been observed in 94% of ovarian cancers and correlates with recurrence and poor prognosis [15]. Growing evidences indicate that STAT3 may represent a promising molecular target of gene therapy for ovarian cancer [29–34]. A variety of therapeutic strategies to block aberrant activity of STAT3 have been developed, such as peptides, oligonucleotides, peptidomimetics, synthetic compounds and natural compounds [35]. Among those inhibitors, natural compounds, for their abundant structural diversity and low toxicity, remain appealing to the field of new drug discovery. Most recently, Zongyuan Yang et al. reported that quercetin, one of the most common flavonoid in nature, enhance dcDDP cytotoxicity in OC with a mechanism involving STAT3 signaling [3]. Another natural compound, arctigenin, has also been shown to be effective in promoting apoptosis in OC cells. Our results suggest that a new withanolide, Physapubescin B, could directly inhibit STAT3 activation and thereby induce cell growth arrest, apoptosis and impede the invasion of OC cells. Additionally, our results also indicate that Physapubescin B could circumvent resistance of OC cells with little toxicity. In conclusion, our study indicates that Physapubescin B was a promising candidate for OC treatment and maybe served as an alternative chemotherapy for patients with advanced disease or recurrence.

## MATERIALS AND METHODS

### Cell culture, plasmids, reagents and transfection

The ES-2, A2780 and A2780/TR cells were cultivated in McCoy's 5a Medium Modified or 1640 Medium plus 2mM L-Glutamine containing 10% fetal bovine serum (FBS). All cells were splitted before confluence and incubated at 37°C in a humidified incubator with 5% CO_2_. pLucTKS3, pLucSRE or β-casein promoter-driven Luc were purchased from addgene. Physapubescin B was purchased from Shanghai GenePharma (Shanghai, China). Transfections were carried out using Lipofectamine-2000 (Invitrogen, Carlsbad, CA) according to the manufacturer’s instructions.

### RNA extraction and qPCR analysis

Total RNA was extracted from cells using Trizol (QIAGEN, Du¨ sseldorf, Germany). cDNA was synthesized with ReverTra Plus (TOYOBO, Japan). The cycling conditions were 10 min at 65 °C for RNA and 60 min at 37 °C for the mix. Quantitative PCR was performed on an ABI 7500 thermocycler (Applied Biosystems) using SYBR® Premix Ex Taq™ (Perfect Real Time) Kits (TaKaRa, Japan) according to the manufacturer’s instructions. Primers used in the assay were listed below:

IL6: Forward 5′-GAGAAAGGACATGTAACAAGAGT-3′
Reverse 5′-GCGCAGAATGAGATGAGTTGT-3′
IL6R: Forward 5′-CTCCTGCCAGTTAGCAGTCC-3′
Reverse 5′- TCTTGCCAGGTGACACTGAG-3′

### Detection of cell proliferation capacity

To determine the cell proliferation capacity, cells were examined with cell growth curve and colony formation assay. Cells were seeded in triplicate in 24-well plates with 1×10^4^ cells per well and were counted over a 3-day period starting from the second day. Cell numbers were determined by a CCK8 cell counting kit according to manufacture’s instructions. The growth curves were drawn for 6 days according to the mean values of the three wells. The number of viable cell colonies was determined 14 days after inoculation of 1×10^3^ cells per well in 12-well plates during colony formation assay. Colony formation ratio was calculated with the following equation: colony formation ratio (%) = (number of colonies/number of seeded cells) × 100.

### Cell invasion assay

A total of 5×10^4^ cells (in 0.2 ml RPMI 1640 with 5% FBS) were seeded into the upper part of a Transwell chamber (Corning, USA), which was pre-coated with 1 mg/ml Matrigel (Growth Factor Reduced BD Matrigel™ Matrix) for 2h. In the lower part of the chamber, 0.6 ml RPMI 1640 with 20% FBS was added. After incubating for 30 h, chambers were disassembled and the membranes were stained with 2% crystal violet for 10 min and placed on a glass slide. Then cells invasing across the membrane were counted in 5 random visual fields under a light microscope. All assays were performed in triplicate and independently performed three times.

### Dual fluorescence reporter assay

cells were seeded in a 48-well plate the day before transfection. The ES-2 cells were cotransfected with pLucTKS3, pLucSRE or β-casein promoter-driven luciferase reporters together with a plasmid expressing the v-Src oncoprotein, and left untreated (0.05% DMSO) or treated with Physapubescin B as indicated. The cells were 24h later and the luciferase intensity was measured with a Dual Luciferase Assay System (Promega) in accordance with the manufacturer’s protocol.

### Western blotting (WB)

Total protein from cells were lyzed by RIPA buffer. The GAPDH was regarded as the endogenous normalizer. The polyclonal rabbit anti-human Cdk2 (sc-748), Cyciln A (sc-596), Cyciln D1 (sc-717) (Santa Cruz, USA) and mouse monoclonal anti-human p21 (sc-817), p53 (sc-47698), Bcl-2 (sc-509), Bcl-xL (sc-136132), Bax (sc-6236), caspase3 (sc-136219), caspase7 (sc-81654), caspase9 (sc-56073), E-cadherin (sc-71009), snail (sc-393172), Vimentin (sc-73259), survivin (sc-101433), STAT3 (sc-293151), p-STAT3 (sc-8001-R), PARP (sc-136208) (Santa Cruz, USA) and GAPDH (SRP00849) (Saierbio, China) antibody were used.

### Immunofluorescence (IF)

Cells were cultured into 70-80% confluence. After fixing and permeabilization, cells were incubated with anti-p-STAT3 (dilution 1:50; Santa Cruz), followed by incubation with tetramethyl rhodamine β-isothiocyanate-conjugated goat anti-mouse IgG (dilution 1:50; Santa Cruz) for 1 h at 25 °C. Nuclei were stained with 100 mg ml−1 4, 6-diamidino-2-phenylindole. Images were acquired with a confocal microscope.

### Electrophoretic mobility shift assay (EMSA)

Forty-eight hours after transfection, cell nuclear extracts were made using a Nuclear Protein Extraction Kit (Fermentas). 10 μg of nuclear extract was incubated with biotin labeled oligonucleotides corresponding to STAT3-binding sequence in gel shift binding buffer (10% glycerol, 20 mM HEPES, pH 7.5, 25 mM KCl, 2 mM DTT, 2 mM MgCl_2_, 0.2% NP40, 1 mg poly(dI-dC). Unlabeled oligonucleotide was added in 200-fold excess as specific competitor. The DNA-protein complexes were then resolved by LightShift® Chemiluminescent EMSA Kit (Pierce) according to the manufacturer’s instructions. The biotin-labeled STAT3-binding DNA probe sequence: 5′-TTCCGGGAA-3′.

### Cell-cycle assay

Transfected ES-2 or A2780 cells were seeded into 6-well plates for 24h in complete medium before cells were deprived of serum for 48h and then returned to complete medium for an additional 24h. All cells were collected by centrifugation, fixed in 95% ethanol, incubated at -20°C overnight and washed with phosphate buffered saline (PBS). Then, cells were resuspended in 1ml FACS solution (PBS, 0.1% TritonX-100, 60 ug/ml propidium iodide (PI), 0.1 mg/ml DNase free RNase, and 0.1% trisodium citrate). After a final incubation on ice for 30 min, cells were analyzed using a FACS Calibur flow cytometer (Beckman Coulter). A total of 10,000 events were counted for each sample.

### Apoptosis detection

Cells were seeded into 6-well plates for 24h in complete medium and then cells were treated with Physapubescin B for 24 or 48h or left untreated. All cells were collected by centrifugation, and then stained with 0.5μg of annexin V for 20 min in the dark and then incubated with 10μg of PI for 5 min, cells were then analyzed using a FACS Calibur flow cytometer (Beckman Coulter). A total of 10,000 events were counted for each sample.

### Statistical analysis

Student′s *t*-test or two-way ANOVA were performed to analyze the significance of differences between the sample means obtained from three independent experiments.

## SUPPLEMENTARY MATERIALS FIGURES



## References

[1] Marchetti C, Palaia I, De Felice F, Musella A, Donfracesco C, Vertechy L, Romito A, Piacenti I, Musio D, Muzii L, Tombolini V, Benedetti Panici P (2016). Tyrosine-kinases inhibitors in recurrent platinum-resistant ovarian cancer patients. Cancer Treat Rev.

[2] Siegel R, Naishadham D, Jemal A (2012). Cancer statistics, 2012. CA Cancer J Clin.

[3] Yang Z, Liu Y, Liao J, Gong C, Sun C, Zhou X, Wei X, Zhang T, Gao Q, Ma D, Chen G (2015). Quercetin induces endoplasmic reticulum stress to enhance cDDP cytotoxicity in ovarian cancer: involvement of STAT3 signaling. FEBS J.

[4] Ramasubbaiah R, Perkins SM, Schilder J, Whalen C, Johnson CS, Callahan M, Jones T, Sutton G, Matei D (2011). Sorafenib in combination with weekly topotecan in recurrent ovarian cancer, a phase I/II study of the Hoosier Oncology Group. Gynecol Oncol.

[5] Korkmaz T, Seber S, Basaran G (2016). Review of the current role of targeted therapies as maintenance therapies in first and second line treatment of epithelial ovarian cancer; in the light of completed trials. Crit Rev Oncol Hematol.

[6] Pisano C, Bruni GS, Facchini G, Marchetti C, Pignata S (2009). Treatment of recurrent epithelial ovarian cancer. Ther Clin Risk Manag.

[7] Takeda K, Noguchi K, Shi W, Tanaka T, Matsumoto M, Yoshida N, Kishimoto T, Akira S (1997). Targeted disruption of the mouse Stat3 gene leads to early embryonic lethality. Proc Natl Acad Sci U S A.

[8] Johnston PA, Grandis JR (2011). STAT3 signaling: anticancer strategies and challenges. Mol Interv.

[9] Darnell JE, Kerr IM, Stark GR (1994). Jak-STAT pathways and transcriptional activation in response to IFNs and other extracellular signaling proteins. Science.

[10] Yu H, Pardoll D, Jove R (2009). STATs in cancer inflammation and immunity: a leading role for STAT3. Nat Rev Cancer.

[11] Yu H, Jove R (2004). The STATs of cancer--new molecular targets come of age. Nat Rev Cancer.

[12] Lu Y, Zhou J, Xu C, Lin H, Xiao J, Wang Z, Yang B (2008). JAK/STAT and PI3K/AKT pathways form a mutual transactivation loop and afford resistance to oxidative stress-induced apoptosis in cardiomyocytes. Cell Physiol Biochem.

[13] Gest C, Mirshahi P, Li H, Pritchard LL, Joimel U, Blot E, Chidiac J, Poletto B, Vannier JP, Varin R, Mirshahi M, Cazin L, Pujade-Lauraine E (2012). Ovarian cancer: Stat3, RhoA and IGF-IR as therapeutic targets. Cancer Lett.

[14] Yang F, Van Meter TE, Buettner R, Hedvat M, Liang W, Kowolik CM, Mepani N, Mirosevich J, Nam S, Chen MY, Tye G, Kirschbaum M, Jove R (2008). Sorafenib inhibits signal transducer and activator of transcription 3 signaling associated with growth arrest and apoptosis of medulloblastomas. Mol Cancer Ther.

[15] Rosen DG, Mercado-Uribe I, Yang G, Bast RC, Amin HM, Lai R, Liu J (2006). The role of constitutively active signal transducer and activator of transcription 3 in ovarian tumorigenesis and prognosis. Cancer.

[16] Levy DE, Darnell JE (2002). Stats: transcriptional control and biological impact. Nat Rev Mol Cell Biol.

[17] Suh YA, Jo SY, Lee HY, Lee C (2015). Inhibition of IL-6/STAT3 axis and targeting Axl and Tyro3 receptor tyrosine kinases by apigenin circumvent taxol resistance in ovarian cancer cells. Int J Oncol.

[18] Tang YJ, Sun ZL, Wu WG, Xing J, He YF, Xin DM, Han P (2015). Inhibitor of signal transducer and activator of transcription 3 (STAT3) suppresses ovarian cancer growth, migration and invasion and enhances the effect of cisplatin *in vitro*. Genet Mol Res.

[19] Shen B (2015). A new golden age of natural products drug discovery. Cell.

[20] Chen LX, He H, Qiu F (2011). Natural withanolides: an overview. Nat Prod Rep.

[21] Reyes-Reyes EM, Jin Z, Vaisberg AJ, Hammond GB, Bates PJ (2013). Physangulidine A, a withanolide from Physalis angulata, perturbs the cell cycle and induces cell death by apoptosis in prostate cancer cells. J Nat Prod.

[22] Ji L, Jin Z, Vaisberg AJ, Hammond GB, Bates PJ (2013). Induction of quinone reductase (QR) by withanolides isolated from Physalis pubescens L. (Solanaceae). Steroids.

[23] Ding W, Hu Z, Zhang Z, Ma Q, Tang H, Ma Z (2015). Physapubescin B exhibits potent activity against human prostate cancer *in vitro* and *in vivo*. J Agric Food Chem.

[24] Barre B, Vigneron A, Perkins N, Roninson IB, Gamelin E, Coqueret O (2007). The STAT3 oncogene as a predictive marker of drug resistance. Trends Mol Med.

[25] Han Z, Feng J, Hong Z, Chen L, Li W, Liao S, Wang X, Ji T, Wang S, Ma D, Chen G, Gao Q (2013). Silencing of the STAT3 signaling pathway reverses the inherent and induced chemoresistance of human ovarian cancer cells. Biochem Biophys Res Commun.

[26] Damu AG, Kuo PC, Su CR, Kuo TH, Chen TH, Bastow KF, Lee KH, Wu TS (2007). Isolation, structures, and structure - cytotoxic activity relationships of withanolides and physalins from Physalis angulata. J Nat Prod.

[27] Mesaik MA, Zaheer-Ul-Haq Murad S, Ismail Z, Abdullah NR, Gill HK, Atta-Ur-Rahman Yousaf M, Siddiqui RA, Ahmad A, Choudhary MI (2006). Biological and molecular docking studies on coagulin-H: human IL-2 novel natural inhibitor. Mol Immunol.

[28] Wake MS, Watson CJ (2015). STAT3 the oncogene - still eluding therapy?. FEBS J.

[29] Zhong LX, Li H, Wu ML, Liu XY, Zhong MJ, Chen XY, Liu J, Zhang Y (2015). Inhibition of STAT3 signaling as critical molecular event in resveratrol-suppressed ovarian cancer cells. J Ovarian Res.

[30] Wen W, Wu J, Liu L, Tian Y, Buettner R, Hsieh MY, Horne D, Dellinger TH, Han ES, Jove R, Yim JH (2015). Synergistic anti-tumor effect of combined inhibition of EGFR and JAK/STAT3 pathways in human ovarian cancer. Mol Cancer.

[31] Ma Y, Zhang X, Xu X, Shen L, Yao Y, Yang Z, Liu P (2015). STAT3 decoy oligodeoxynucleotides-loaded solid lipid nanoparticles induce cell death and inhibit invasion in ovarian cancer cells. PLoS One.

[32] Wen W, Liang W, Wu J, Kowolik CM, Buettner R, Scuto A, Hsieh MY, Hong H, Brown CE, Forman SJ, Horne D, Morgan R, Wakabayashi M (2014). Targeting JAK1/STAT3 signaling suppresses tumor progression and metastasis in a peritoneal model of human ovarian cancer. Mol Cancer Ther.

[33] Rath KS, Naidu SK, Lata P, Bid HK, Rivera BK, McCann GA, Tierney BJ, ElNaggar AC, Bravo V, Leone G, Houghton P, Hideg K, Kuppusamy P (2014). HO-3867, a safe STAT3 inhibitor, is selectively cytotoxic to ovarian cancer. Cancer Res.

[34] Liu M, Naidu SK, Lata P, Bid HK, Rivera BK, McCann GA, Tierney BJ, Elnaggar AC, Bravo V, Leone G, Houghton P, Hideg K, Kuppusamy P (2014). Blockage of STAT3 signaling pathway with a decoy oligodeoxynucleotide inhibits growth of human ovarian cancer cells. Cancer Invest.

[35] Masciocchi D, Gelain A, Villa S, Meneghetti F, Barlocco D (2011). Signal transducer and activator of transcription 3 (STAT3): a promising target for anticancer therapy. Future Med Chem.

