# Supplementary material for: Physapubescin B inhibits tumorgenesis and circumvents taxol resistance of ovarian cancer cells through STAT3 signaling
Source: Oncotarget. 2017 Jul 26;8(41):70130–41. doi: 10.18632/oncotarget.19593 (PMC5642541; doi:10.18632/oncotarget.19593)
Supplement: Supplementary file 1 [file oncotarget-08-70130-s001.pdf]

Physapubescin B inhibits tumorgenesis and circumvents taxol resistance of ovarian cancer cells through STAT3 signaling

SUPPLEMENTARY MATERIALS

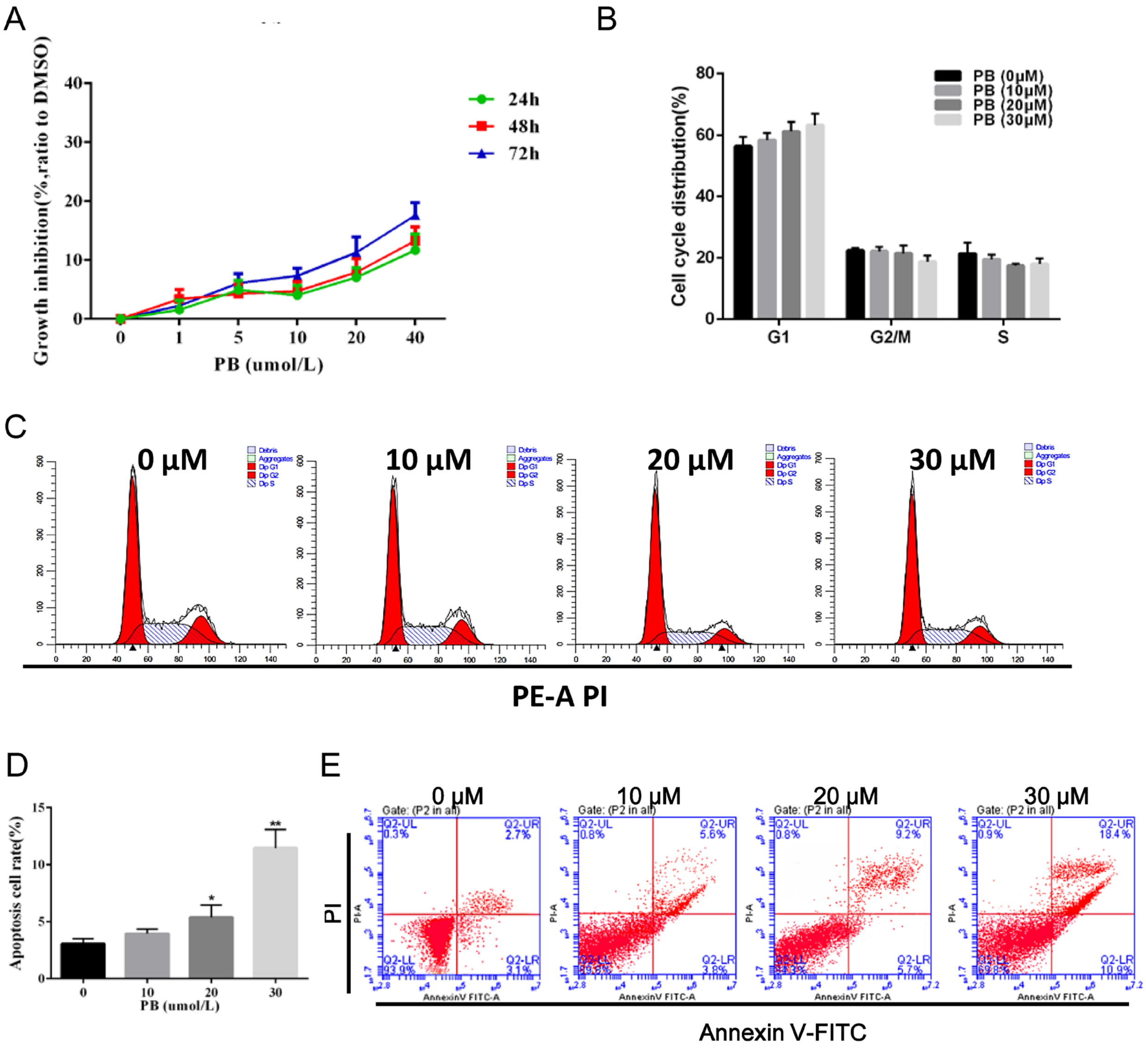

Supplementary Figure 1: Effects of Physapubescin B on HOSE cells. **(A)** Different dosages of Physapubescin B were added into cell cultures. Cell viabilities of HOSE cells were determined by CCK8 assays at each time points as indicated. **(B and C)** Cell cycles were checked by PI staining, 24 h after Physapubescin B treatment. (C) Statics results and (D) representative pictures. **(D and E)** Apoptosis of HOSE cells treated by different concentrations of Physapubescin B as indicated for 24h were analyzed by FACS. (A) Representative pictures and (B) stastics results. Data are presented as mean±SD. **P*<0.05, ***P*<0.01. All experiments were repeated at least three times independently.

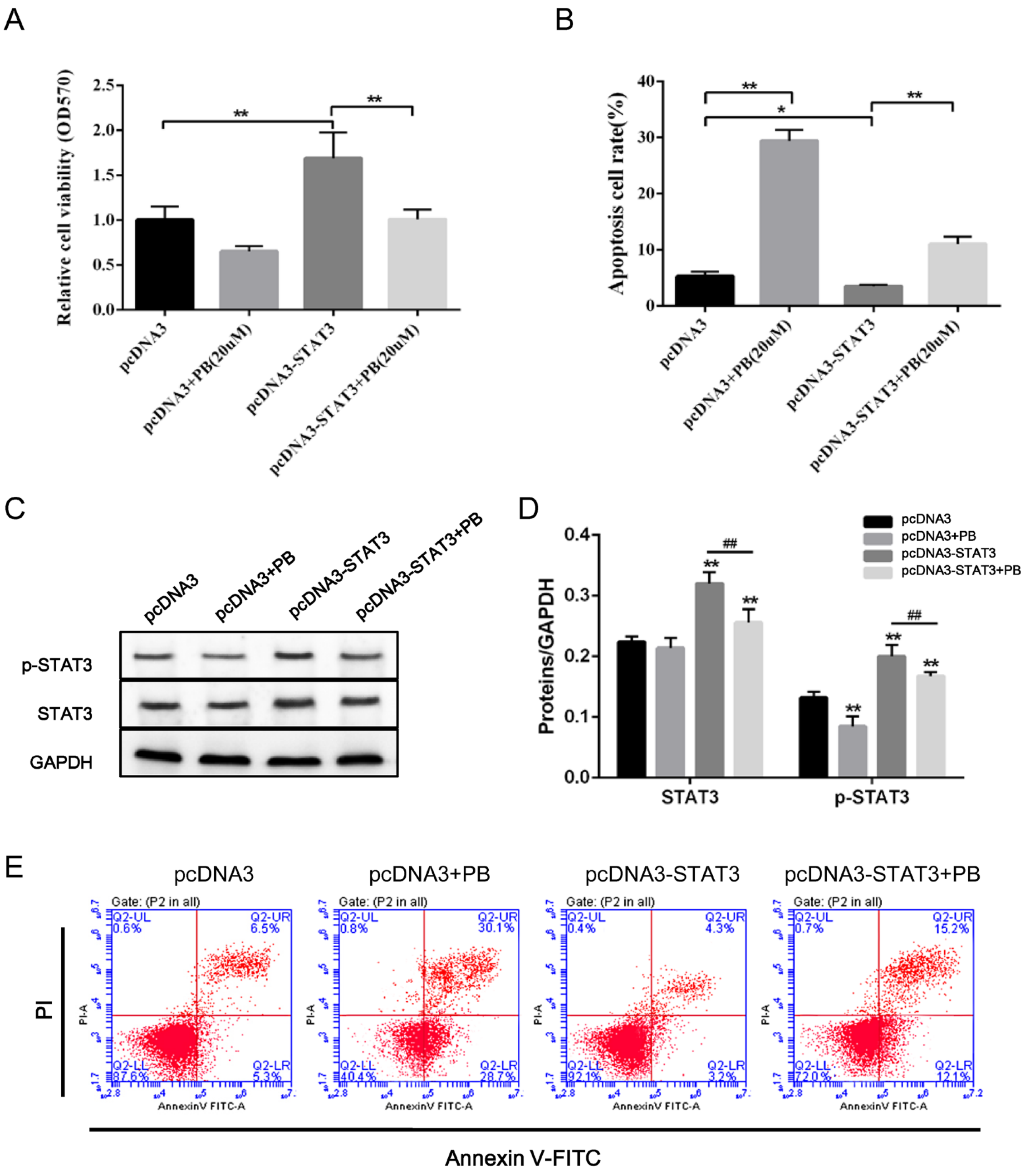

Supplementary Figure 2: The effects of Physapubescin B were partially dependent on STAT3. ES-2 cells were transfected with STAT3 or pCDNA3 vector. 24 h later, cells were treated with Physapubescin B (20 μmol/L) or left untreated for 48 h. **(A)** Cell viabilities were determined by CCK8 assays. **(B and E)** Apoptosis were analyzed by FACS. (B) Statics results and (E) representative pictures. **(C and D)** Whole cell lysates were subjected to western bolt analysis. GAPDH served as a loading control. (C) representative pictures and (D) results of gray-scale scanning. Data are presented as mean±SD. **P*<0.05, ***P*<0.01. All experiments were repeated at least three times independently.
